# 3', 4'-dihydroxyflavone ameliorates paclitaxel model of peripheral neuropathy in mice by modulating K_ATP_ channel, adenosine (A_3_) and GABA_A_ (α_2_ subunit) receptors

**DOI:** 10.6026/97320630019754

**Published:** 2023-06-30

**Authors:** Kavitha Ramasamy, Jaikumar Shanmugasundaram, Viswanathan Subramanian, Rajesh Manoharan, Parimala Kathirvelu, Rajagopalan Vijayaraghavan

**Affiliations:** 1 Department of Pharmacology, Sri Ramachandra Medical College and Research Institute, Sri Ramachandra Institute of Higher Education and Research, Chennai - 600116, India; 2 Department of Pharmacology, Panimalar Medical College Hospital and Research Institute, Chennai - 600123, India; 3 Department of Pharmacology, Meenakshi Medical College Hospital and Research Institute, Meenakshi Academy of Higher Education and Research, Kanchipuram - 631552, India; 4 Department of Pharmacology, Sri Muthukumaran Medical College and Research Institute, Chennai - 600069, India; 5 Director Research, Saveetha Institute of Medical And Technical Sciences, Thandalam, Chennai - 602105, India

**Keywords:** Paclitaxel, 3', 4'-dihydroxyflavone, CIPN, GABA_A_ (α_2_ subunit), adenosine (A_3_), K_ATP_ channels

## Abstract

Paclitaxel is a widely used cancer chemotherapeutic agent for many solid tumors; but peripheral neuropathy is a major limitation for its clinical use. Studies have demonstrated the usefulness of flavone derivatives in chemotherapy induced peripheral neuropathy. The present study evaluates the anti-neuropathic effect of 3', 4'-dihydroxyflavone on paclitaxel-induced peripheral neuropathy and the underlying mechanisms. Paclitaxel was administered to mice in a single dose of 10 mg/kg, i.p.The neuropathic behavioural parameters such as mechanical allodynia, cold allodynia and thermal hyperalgesia were assessed 24 h later. The test compound 3', 4'-dihydroxyflavone (50,100 or 200 mg/kg,s.c) was administered 30 min prior to the assessment of behavioral parameters. The possible mechanisms involving K_ATP_ channels, adenosine and GABA_A_ receptors were explored by employing suitable interacting drugs. Molecular docking studies to predict the binding interactions of 3', 4'-dihydroxyflavone at the above targets were also carried out. The test compound 3', 4'-dihydroxyflavoneexhibited a significant reduction in paw withdrawal response score in both mechanical and cold allodynia and also increased the tail flick response time in thermal hyperalgesia due to paclitaxel-induced neuropathy. The anti-neuropathic effect of 3', 4'-dihydroxyflavonewas significantly reversed by pre-treatment with glibenclamide, caffeine or bicuculline revealing the involvement of K_ATP_ channels, adenosine and GABA_A_ receptors respectively. Furthermore, the molecular docking studies indicated a favourable binding affinity and good H-bond interaction of 3', 4'-dihydroxyflavone at these targets. The findings of the present study suggests that, 3', 4'-dihydroxyflavone has anti-neuropathic effect against paclitaxel-induced peripheral neuropathy through mechanisms that involve K_ATP_ channels, adenosine (A_3_) and GABA_A_ (α_2_ subunit) receptors.

## Background:

Chemotherapy-induced peripheral neuropathy (CIPN)is a severe debilitating consequence of many first line anticancer drugs like platinum analogues, taxanes, vinca alkaloids and proteasome inhibitors [1].Taxanes are a class of diterpenoids which include paclitaxel, docetaxel and cabazitaxel that effectively prevent cancer proliferation by stabilising microtubules, resulting in cell cycle arrest and aberrant mitosis[2]. They have been used as frontline anticancer drugs in many of the solid tumours in breast, ovary, lung, pancreas and prostate. Paclitaxel is the prototype of taxane family of anticancer drugs and most commonly used. Peripheral neuropathy is the most prevalent dose limiting adverse effect of taxanes affecting up to 97% of patients treated with paclitaxel and becomes persistent in over 60% of the cases[3].Paclitaxel induced peripheral neuropathy is predominately sensory neuropathy characterised by numbness, tingling, spontaneous pain and evoked pain to mechanical and cold stimuli and patients frequently report a stocking and glove distribution [4].The severity of pain may necessitate a reduction in dose or abrupt withdrawal of chemotherapy which can affect tumour control and survival besides adversely impacting the quality of life of the patient. The underlying mechanism of paclitaxel induced peripheral neuropathy remains unclear and recent evidences demonstrate a combination of axonal degeneration, oxidative stress, ion channel dysregulation and inflammatory events in the development of neuropathy[5].Therefore, the investigation of an agent interfering in the above pathogenesis is a potential strategy to prevent or treat CIPN.The currently recommended treatment regimen for CIPN include duloxetine, tricyclic antidepressants, anticonvulsants, compounded topical products, NSAID and opioid therapy. However, these drugs have inherent adverse effects and hence, there is an imminent need for the development of a novel compound with promising anti-neuropathic effect in alleviating CIPN. Polyphenolic compounds such as flavone derivatives have been extensively studied for various pharmacological actions and therapeutic applications in many diseases. Studies have demonstrated the potent antioxidant [6], anti-nociceptive [7] and neuroprotective [8] effects of flavone derivatives. The aforementioned actions of flavones may exert a protective effect in the pathogenesis of CIPN suggesting that, flavone derivatives may be considered as suitable candidates to treat CIPN.A few flavone derivatives have been found to attenuate the symptoms that develop in paclitaxel model of peripheral neuropathy[9,10].In a recent study,7,3'-dihydroxyflavone has been reported to exert a protective effect against paclitaxel induced neuropathy in mice involving GABA_A_, adenosine receptors and K_ATP_ channel [11]. Apart from this report, literature evidences are sparse on the effect of dihydroxyflavones on CIPN. In an earlier study, 3',4'-dihydroxyflavone was found to significantly attenuate acetic acid induced abdominal constrictions in mice involving opioid mechanism [12]. Hence, it was considered interesting to investigate the anti-neuropathic effect of 3',4'-dihydroxyflavone against paclitaxel-induced peripheral neuropathy in mice. It is pertinent to mention that, flavone derivatives have been found to interact with several neurotransmitters and ion channels which are implicated in CIPN. Recently, studies have reported the anti-neuropathic effect of a synthetic flavone involving a2 subunit containing GABA_A_ receptors in cisplatin model of peripheral neuropathy in mice [13]. Studies have also demonstrated the involvement of ion channels such as K_V_ and K_ATP_ channels in the induction of peripheral neuropathy with paclitaxel [14]. Moreover, activation of adenosine A3 receptors has been shown to exert an anti-allodynic effect in CIPN [15]. Hence, the present investigation aims to evaluate the potential anti-neuropathic effect of 3',4'-dihydroxyflavone against paclitaxel model of peripheral neuropathy in mice by evaluating mechanical allodynia, cold allodynia and thermal hyperalgesia utilizing a battery of tests such as von Frey's hair aesthesiometer, acetone bubble and hot water tail immersion tests respectively. In addition, suitable interacting chemicals have been administered to ascertain the involvement of GABA_A_ receptor, adenosine receptor and K_ATP_ channel in the neuroprotective effect of 3',4'-dihydroxyflavone.Molecular docking studies of a ligand with the target receptor protein is considered a valuable tool to predict the type of interaction of a ligand at the receptor site and may offer additional confirmation to the evidences observed in *in vivo* studies. Hence, molecular docking studies have also been performed to predict the binding sites and H-bond interactions of 3',4'-dihydroxyflavone at these targets.

## Materials and Methods:

## Animals:

The behavioural experiments were conducted in Swiss albino mice of either sex (25-30 g). The animals were placed in polypropylene cages with soft bedding, free access to standard pellet diet and water under environmentally controlled conditions (22 ± 2°C, 12 h light/12 h dark cycle, lights on at 7 a.m.). The mice were randomly selected for each test group and consist of a minimum of six animals. To avoid circadian variations and also to maintain uniformity, the behavioural experiments were conducted between 09:00 h and 14:00 h. The experiments were carried out with the approval of the institutional animal ethics committee (IAEC No. 005/2019). The guidelines prescribed by the committee for the purpose of control and supervision of experiments on animals (CPCSEA) New Delhi, India, regarding the care and handling of animals were meticulously followed during the experimental procedures.

## Drugs and Chemicals:

**Figure 1:**
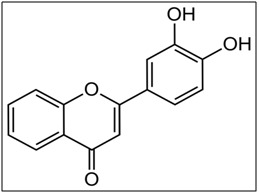
Chemical structure of 3', 4'-dihydroxyflavone

Peripheral neuropathy in mice was induced with paclitaxel (Intas, India) diluted in physiological saline and administered as a single dose of 10 mg/kg, i.p. A fine suspension of the test compound, 3',4'-dihydroxyflavone (3',4'-DHF, Figure 1; Research organics, Chennai, India) was prepared in 0.5% carboxy methylcellulose (CMC) and administered subcutaneously (s.c) to animals 30 min prior to the experimental procedure. The standard drug, gabapentin (Tokyo Chemical Industry Co Ltd., Japan) was dissolved in physiological saline before administration (70 mg/kg,i.p). In mechanism studies, the following interacting chemicals have been administered by intraperitoneal (i.p.) route. A fine suspension of (+) Bicuculline (Tokyo Chemical Industry Co Ltd., Japan), GABA_A_ receptor antagonist (2 mg/kg) was prepared in 2% Tween-80.Caffeine (Himedia, India) in a dose of 50 mg/kg, dissolved in physiological saline was administered as a non-selective antagonist at adenosine receptors. AK_ATP_ channel blocker, glibenclamide (Dr.Reddy's Laboratory, India) was prepared in 0.5% CMC and administered in a dose of 10 mg/kg. The above drugs were freshly prepared on the day of the experiment and administered by s.c / i.p route in a volume of 10 ml/kg body weight.

## Induction of peripheral neuropathy by paclitaxel:

Different groups of mice (n = 6or7/group) were administered with paclitaxel (10 mg/kg, i.p) on day one. One group of animals received the vehicle (0.5% CMC) alone. To confirm the neuropathic manifestations, mice were evaluated for behavioural parameters such as mechanical allodynia, cold allodynia and thermal hyperalgesia 24 h after administration of paclitaxel [16]. On day two, the animals that received paclitaxel were administered with the following drug treatments (*viz*): vehicle, gabapentin (70 mg/kg, i.p) or 3',4'-dihydroxyflavone (50, 100 or 200 mg/kg, S.C) 30 min prior to the behavioural experiments. An observer blinded to the treatment schedule assessed the scores in the behavioural tests. The doses of 3',4'-dihydroxyflavone were selected based on its anti-nociceptive action [12].

##  Behavioural assessment:

## Evaluation of mechanical allodynia in mice (von Frey's Hair aesthesiometer test):

The test involves the exploration of dynamic responses towards a tactile stimulus. The apparatus consists of an inverted transparent plastic box (13x7x7 cm) secured on a raised steel frame with the floor made of wire mesh. After10 min of habituation period, von Frey filament measuring 15 mm length was applied five times in a perpendicular direction against the mid-plantar skin of both hind paws of mice at an interval of 30 sec. The paw withdrawal response was scored as: 0 - no response, 1 - move away from the filament, 2 - immediate flinching or licking of the hind paw [17]. The paw withdrawal response score calculated as the sum of ten values observed from both hind paws was recorded prior to and 30 min after different drug treatments.

## Evaluation of cold allodynia (Acetone bubble test):

The test for cold allodynia was carried out by the method described earlier [18]. Each mice was habituated for 10 min in an inverted transparent plastic box (13x7x7 cm) secured on a raised steel frame with a meshed wire floor. After habituation, 0.05 mL of acetone formed as a bubble at the tip of a one mL syringe was applied three times alternatively to the mid-plantar surface of both hind pawsat an interval of 1 min. The paw withdrawal response was observed for a period of 20 sec and the score was recorded prior to and 30 min after drug treatments. The paw withdrawal responses have been graded as follows: 0 - no response, 1 - immediate withdrawal, 2 - prolonged withdrawal and 3 - licking / biting of the hind paw. The sum of six values obtained from both hind paws was taken as the paw withdrawal response score.

## Evaluation of thermal hyperalgesia (Hot water tail immersion test):

Hot water tail immersion test as described earlier [19] was employed to assess thermal hyperalgesia. After each mouse was restrained in a mouse holder, the tip of the mouse tail (2-3 cm) was immersed in hot water bath maintained at 48 ± 0.5°C. The reaction time taken to withdraw the tail from the hot water was recorded before and 30 min after drug treatments. To protect the tail from injury, a cut off time of 20 sec was applied. An increase in the reaction time between pre and post drug treatments was indicative of an anti-nociceptive response.

## Investigation of mechanisms involved in the anti-neuropathic effect of 3',4'-dihydroxyflavone:

The schedule of drug treatment and the time for evaluation of behavioural parameters in the mechanism studies is presented in Table 1. On day one, mice that were randomly allocated to different groups (n=6/group) received paclitaxel 10 mg/kg, i.p. On the next day, the animals were pre-treated with either vehicle or one of the interacting drugs after the initial evaluation of behavioural parameters. Mice were treated with vehicle or 3',4'-dihydroxyflavone (200 mg/kg, s.c) 15 min after the administration of interacting chemicals and again subjected to behavioural experiments 30 min later. The doses of the interacting drugs were carefully selected based on earlier reports [11] so that the doses employed were sufficient enough to exert an antagonistic effect but without inducing any adverse effect *per se.*

## Molecular docking studies:

The binding of 3',4'-dihydroxyflavone with targets such as GABA_A_ (α_2_ subunit), K_ATP_ channel and adenosine (A_3_) receptors was carried out using molecular docking as described earlier [20]. The gene coded amino acid sequence of the target proteins were retrieved in FASTA format using NCBI-Gene database and UniProt proteomics database [GABA_A_ α_2_ subunit (P47869), K_ATP_ channel (Q14500) and Adenosine (A_3_) receptor (P0DMS8)]. The amino acid sequences were converted into three dimensional (3-D) structures using automated protein modelling server CPH3.0 model server. The compounds 3',4'-dihydroxyflavone (CID: 145726), adenosine (CID: 60961), GABA (CID: 119) and pinacidil (CID: 4826) were retrieved from NCBI Pub chem compound database. The docked protein structures along with the ligands were viewed using Accelrys Discovery Studio software (2.5.5 v).

## Statistical analysis:

The data obtained from various treatments (vehicle, gabapentin or 3',4'-dihydroxyflavone) in paclitaxel induced mechanical allodynia, cold allodynia and thermal hyperalgesia was statistically analysed by two-way ANOVA followed by *post hoc* Bonferroni multiple comparison test to compare between multiple groups. The data collected from mechanism studies was subjected to three-way ANOVA followed by *post hoc* Bonferroni multiple comparison test using Sigma Plot version 14.5 (Systat software, San Jose, USA). Results are expressed as mean ± S.E.M. Probability values less than 0.05 were considered as statistically significant.

## Results:

Paclitaxel treated mice exhibited the typical neuropathic manifestations such as mechanical allodynia, cold allodynia and thermal hyperalgesia on the next day. Treatment with different doses of 3',4'-dihydroxyflavone or gabapentin significantly ameliorated the above behavioural responses.

## Effect of 3',4'-dihydroxyflavoneon mechanical allodynia:

**Figure 2:**
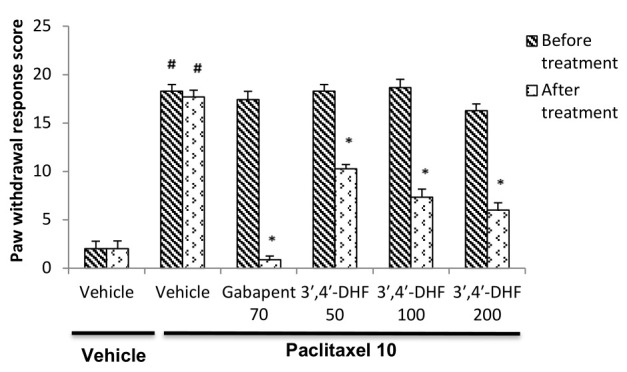
Effect of 3', 4'-dihydroxyflavone (3', 4'-DHF) on paw withdrawal response score in paclitaxel induced mechanical allodynia in mice. Each bar represents mean ± S.E.M (n = 6 or 7). Mice were administered with vehicle / paclitaxel (10 mg/kg, i.p) on the previous day. Experiments were carried out in mice 30 min after treatment with vehicle, gabapentin (70 mg/kg) or 3', 4'-dihydroxyflavone (50, 100 or 200 mg/kg) on next day. Statistical analysis was performed by two-way ANOVA followed by post hoc Bonferroni test for multiple comparisons. * p < 0.001 compared to the value before respective treatment. # p < 0.001 compared to vehicle-vehicle treatment group.

In paclitaxel treated animals, application of von Frey's filament on the plantar surface of the hind paw resulted in an aversive behavioural responses such as flinching or moving away from the filament that was graded and scored. The vehicle treated mice did not show any alterations in the paw withdrawal response score (Figure 2). In paclitaxel treated animals significant increase in the paw withdrawal response score (P < 0.001) was recorded compared to vehicle-vehicle treated group, thus indicating the induction of neuropathic symptoms. Treatment with3',4'-dihydroxyflavone revealed a significant anti-neuropathic effect comparable to gabapentin against paclitaxel model of mechanical allodynia. Two-way ANOVA showed significant decrease in the paw withdrawal score with different drug treatments [F (5, 70) = 119.20, p < 0.001] and also between pre and post treatment values [F (5, 70) = 41.83, p < 0.001]. Subsequent *post hoc* analysis with Bonferroni test showed significant decrease in the paw withdrawal score between pre and post treatment observations in mice treated with gabapentin (p < 0.001) or different doses of 3',4'-dihydroxyflavone (p < 0.001) when compared to paclitaxel + vehicle treated animals (Figure 2).

## Effect of 3',4'-dihydroxyflavoneonthermal hyperalgesia:

In paclitaxel treated animals a significant reduction in the tail flick response time (p < 0.001) compared to vehicle-vehicle treated group confirms the development of thermal hyperalgesia in mice (Figure 4). Treatment with 3',4'-dihydroxyflavone and gabapentin revealed significant anti-hyperalgesic effect against paclitaxel model of thermal hyperalgesia. Two-way ANOVA showed significant increase in the tail flick reaction time with various drug treatments [F (5, 70) = 23.23, p < 0.001] and also between pre and post treatment observations [F (5, 70) = 20.84, p < 0.001]. Bonferroni *post hoc* analysis revealed a significant increase in the tail flick reaction time between pre and post treatment values in mice treated with different doses of 3',4'-dihydroxyflavone(p < 0.001) or the standard drug gabapentin (p < 0.001) when compared to paclitaxel + vehicle treated animals (Figure 4).

## Effect of 3',4'-dihydroxyflavoneoncold allodynia:

**Figure 3:**
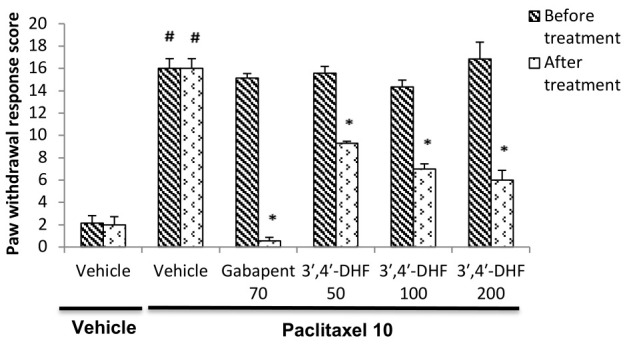
Effect of 3', 4'-dihydroxyflavone (3', 4'-DHF) on paw withdrawal response score in paclitaxel induced cold allodynia in mice. Each bar represents mean ± S.E.M (n = 6 or 7). Mice were administered with vehicle / paclitaxel (10 mg/kg, i.p) on the previous day. Experiments were carried out in mice 30 min after treatment with vehicle, gabapentin (70 mg/kg) or 3', 4'-dihydroxyflavone (50, 100 or 200 mg/kg) on next day. Statistical analysis was performed by two-way ANOVA followed by post hoc Bonferroni test for multiple comparisons. * p < 0.001 compared to the value before respective treatment. # p < 0.001 compared to vehicle-vehicle treatment group.

**Figure 4:**
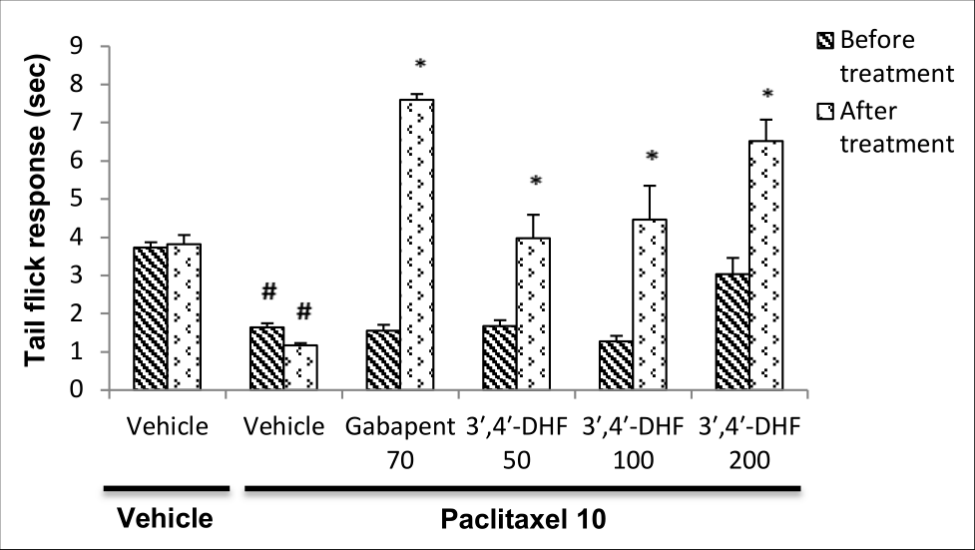
Effect of 3', 4'-dihydroxyflavone (3', 4'-DHF) on tail flick response time in paclitaxel induced thermal hyperalgesia in mice. Each bar represents mean ± S.E.M (n = 6 or 7). Mice were administered with vehicle / paclitaxel (10 mg/kg, i.p) on the previous day. Experiments were carried out in mice 30 min after treatment with vehicle, gabapentin (70 mg/kg) or 3', 4'-dihydroxyflavone (50, 100 or 200 mg/kg) on next day. Statistical analysis was performed by two-way ANOVA followed by post hoc Bonferroni test for multiple comparisons. * p < 0.001 compared to the value before respective treatment. # p < 0.001 compared to vehicle-vehicle treatment group.

An intense aversive response like immediate paw withdrawal or licking / biting behaviour was observed after application of acetone to the plantar surface of hind pawsin paclitaxel administered mice. The mean paw withdrawal response score was unaltered with vehicle treatment. A significant increase in the paw withdrawal response score (p < 0.001) noted in paclitaxel treated group compared to vehicle-vehicle treated animals confirms the development of cold allodynia in mice. The mean paw withdrawal score was unaltered with vehicle treatment. A significant anti-allodynic effect was observed in mice after treatment with 3',4'-dihydroxyflavone in paclitaxel model of cold allodynia (Figure 3). A significant reduction in the paw withdrawal score was identified by two-way analysis of variance with various drug treatments [F (5, 70) = 79.72, p < 0.001] and also between pre and post treatment values [F (5, 70) = 29.86, p < 0.001]. Further Bonferroni *post hoc* analysis showed a significant decrease in the paw withdrawal score between pre and post treatment observations in mice treated with gabapentin (p < 0.001) or different doses of 3',4'-dihydroxyflavone (p < 0.001) when compared to paclitaxel + vehicle treated group (Figure 3).

## Effect of bicuculline pre-treatment on the response to 3',4'-dihydroxyflavone:

**Figure 5:**
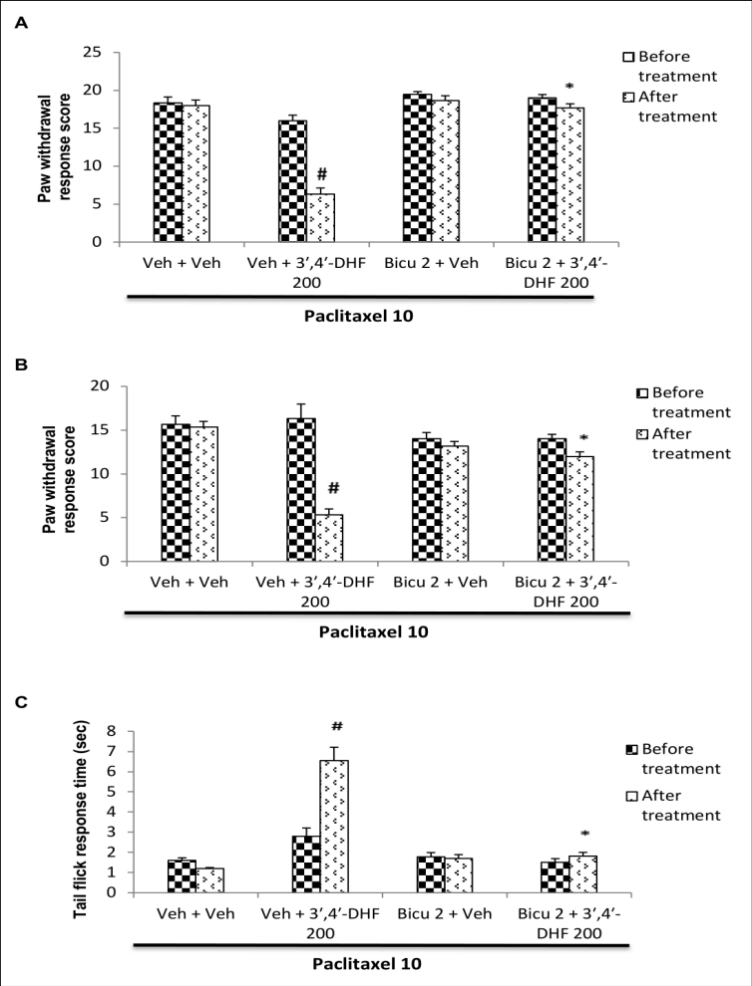
Effect of bicuculline pre-treatment on the response of 3', 4'-dihydroxyflavone (3',4'-DHF) in paclitaxel induced (A) mechanical allodynia (B) cold allodynia and (C) thermal hyperalgesia in mice. Each bar represents mean ± S.E.M (n = 6). Statistical analysis was performed by three-way ANOVA followed by post hoc Bonferroni test for multiple comparisons. * p < 0.001 compared to vehicle + 3',4'-DHF treated group. # p < 0.001 compared to vehicle + vehicle treated group. All treatment groups received paclitaxel (10 mg/kg, i.p) on the previous day. On the next day, different groups of mice were pre-treated with vehicle or bicuculline (2 mg/kg, i.p) and 15 min later received vehicle or 3',4'-DHF (200mg/kg, s.c). Behavioural assessments were made before any drug administration and 30 min after vehicle / 3', 4'-DHF treatment.

The behavioural responses of mice administered with 3',4'-dihydroxyflavone after bicuculline pre-treatment in paclitaxel model of neuropathy is shown in Figure 5. The anti-neuropathic effect observed with 3',4'-dihydroxyflavone treatment in paclitaxel induced neuropathic manifestations was significantly reversed with bicuculline pre-treatment (Figure 5A-C). In paclitaxel administered mice, vehicle or bicuculline treatment per se did not alter the increase in paw withdrawal score observed from mechanical allodynia, cold allodynia or the reduction in the tail flick reaction time due to thermal hyperalgesia. However, the decrease in paw withdrawal score of both mechanical (Figure 5A) and cold allodynia (Figure 5B) and the increase in tail flick reaction time (Figure 5C) observed after 3',4'-dihydroxyflavone treatment was significantly reversed with bicuculline pre-treatment. Three-way ANOVA showed significant interaction between 3',4'-dihydroxyflavone and bicuculline treatment in mechanical allodynia [F (1, 40) = 23.22, p < 0.001], cold allodynia [F (1, 40) = 15.37, p < 0.001] and thermal hyperalgesia [F (1, 40) = 18.10, p < 0.001]. *post hoc* analysis with Bonferroni test demonstrated significant reversal of the responses to 3',4'-dihydroxyflavone with bicuculline pre-treatment (p < 0.001) compared to vehicle + 3',4'-dihydroxyflavone treated group in all the three parameters tested (Figure 5A-C).

## Effect of glibenclamide pre-treatment on the response to 3',4'-dihydroxyflavone:

**Figure 6:**
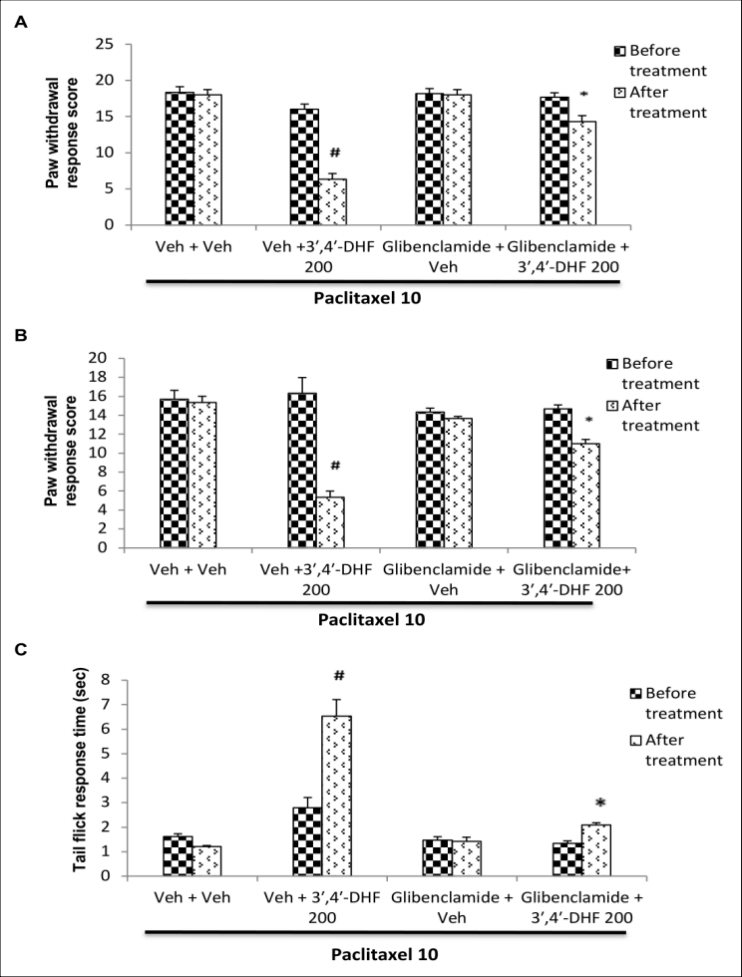
Effect of glibenclamide pre-treatment on the response of 3', 4'-dihydroxyflavone (3',4'-DHF) in paclitaxel induced (A) mechanical allodynia (B) cold allodynia and (C) thermal hyperalgesia in mice. Each bar represents mean ± S.E.M (n = 6). Statistical analysis was performed by three-way ANOVA followed by post hoc Bonferroni test for multiple comparisons. * p <0.001 compared to vehicle + 3',4'-DHF treated group. # p <0.001 compared to vehicle + vehicle treated group. All treatment groups received paclitaxel (10 mg/kg, i.p) on the previous day. On the next day, different groups of mice were pre-treated with vehicle or glibenclamide (10 mg/kg, i.p) and 15 min later received vehicle or 3',4'-DHF (200 mg/kg, s.c). Behavioural assessments were made before any drug administration and 30 min after vehicle / 3', 4'-DHF treatment.

The behavioural responses of mice after administration of 3',4'-dihydroxyflavone with glibenclamide pre-treatment in paclitaxel model of neuropathy is shown in Figure 6. Glibenclamide pre-treated animals significantly reversed the anti-neuropathic effect of 3',4'-dihydroxyflavone against paclitaxel induced neuropathic manifestations (Figure 6A-C). Vehicle or glibenclamide administration per se did not alter the elevated paw withdrawal score due to mechanical and cold allodynia or the reduction in reaction time to flick the tail in thermal hyperalgesia in paclitaxel treated mice. However, pre-treatment with glibenclamide, a K_ATP_ channel blocker, significantly reversed the reduction in paw withdrawal score of both mechanical (Figure 6A) and cold allodynia (Figure 6B) and the increase in tail flick reaction time (Figure 6C) produced by 3',4'-dihydroxyflavone in paclitaxel treated mice. Three way analysis of variance showed significant interaction between 3',4'-dihydroxyflavone and glibenclamide treatment in mechanical allodynia [F (1, 40) = 8.63, p <0.001], cold allodynia [F (1, 40) = 11.50, p <0.001] and thermal hyperalgesia [F (1, 40) = 16.12, p < 0.001]. Further Bonferroni *post hoc* analysis showed significant reversal of the responses to 3',4'-dihydroxyflavone with glibenclamide pre-treatment (p < 0.001) compared to vehicle + 3',4'-dihydroxyflavone treated group (Figure 6A-C).

## Effect of caffeine pre-treatment on the response to 3',4'-dihydroxyflavone:

**Figure 7:**
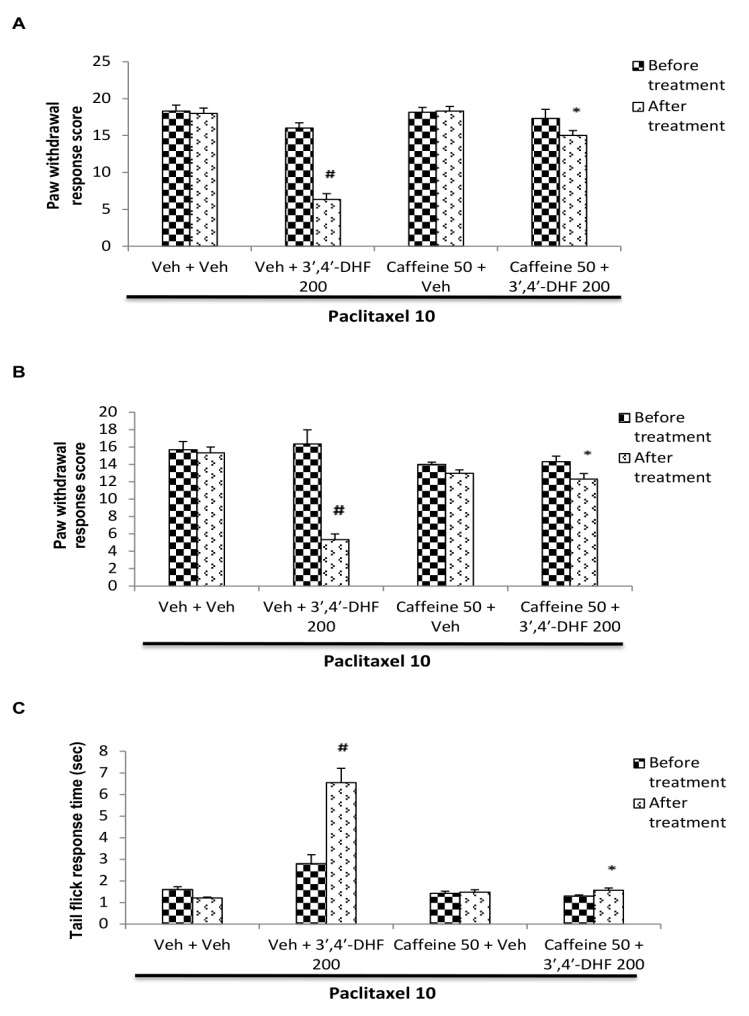
Effect of caffeine pre-treatment on the response of 3', 4'-dihydroxyflavone (3', 4'-DHF) in paclitaxel induced (A) mechanical allodynia (B) cold allodynia and (C) thermal hyperalgesia in mice. Each bar represents mean ± S.E.M (n = 6). Statistical analysis was performed by three-way ANOVA followed by post hoc Bonferroni test for multiple comparisons. * p <0.001 compared to vehicle + 3',4'-DHF treated group. # p <0.001 compared to vehicle + vehicle treated group. All treatment groups received paclitaxel (10 mg/kg, i.p) on the previous day. On the next day, different groups of mice were pre-treated with vehicle or caffeine (50 mg/kg, i.p) and 15 min later received vehicle or 3',4'-DHF (200 mg/kg, s.c). Behavioural assessments were made before any drug administration and 30 min after vehicle / 3', 4'-DHF treatment.

The behavioural responses of 3',4'-dihydroxyflavone-treated mice that have been pre-treated with caffeine in paclitaxel model of neuropathy are shown in Figure 7. Caffeine pre-treatment significantly reversed the anti-neuropathic effect of 3',4'-dihydroxyflavone against paclitaxel induced neuropathic manifestations in mice (Figure 7A-C). In paclitaxel treated animals, vehicle or caffeine administration per se did not alter the elevated paw withdrawal score due to mechanical and cold allodynia or the reduction in reaction time to flick the tail in thermal hyperalgesia. However, pre-treatment with caffeine, an adenosine receptor antagonist, significantly reversed the reduction in paw withdrawal response score of both mechanical (Figure 7A) and cold allodynia (Figure 7B) and the increase in tail flick response time (Figure 7C) produced by 3',4'-dihydroxyflavone in paclitaxel treated mice. Three-way ANOVA showed significant interaction between caffeine and 3',4'-dihydroxyflavone treatment in mechanical allodynia [F (1, 40) = 9.09, p < 0.001], cold allodynia [F (1, 40) = 17.09, p < 0.001] and thermal hyperalgesia [F (1, 40) = 23.08, p < 0.001]. Subsequent Bonferroni *post hoc* analysis revealed significant reversal of the responses to 3',4'-dihydroxyflavone with caffeine pre-treatment (p < 0.001)compared to vehicle + 3',4'-dihydroxyflavone treated group (Figure 7A-C).

## Molecular docking studies:

**Figure 8:**
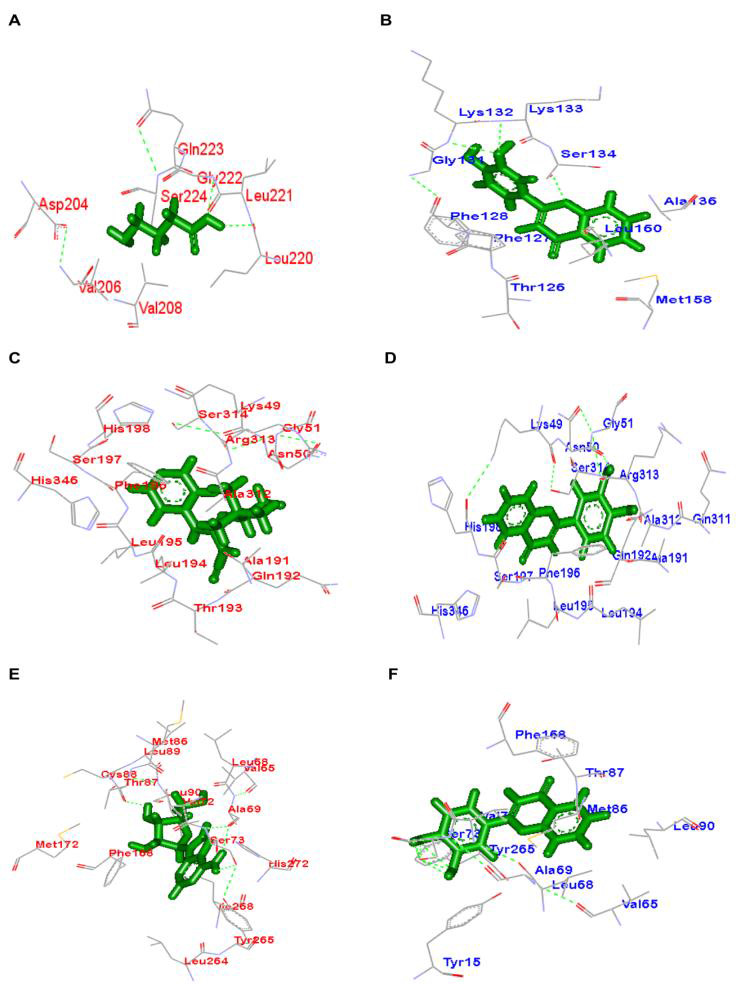
3D model showing binding site of3', 4'-dihydroxyflavone (3', 4'-DHF) and standard ligands (stick model) at GABAA (α_2_ subunit) receptor (wire frame model), KATP channel (wire frame model) and Adenosine (A3) receptor (wire frame model). (A) GABAA receptor + GABA, (B) GABAA receptor + 3',4'-DHF, (C) KATP channel + Pinacidil (D) KATP channel + 3',4'-DHF, (E) Adenosine (A_3_) receptor + adenosine and (F) Adenosine (A3) receptor + 3',4'-DHF. The hydrogen bond interactions of the ligands at GABAA (α_2_ subunit), KATP channel and Adenosine (A_3_) receptor are shown as green dotted lines. The hydrophobic interactions established by these compounds at GABAA (α2 subunit), KATP channel and Adenosine (A3) receptor are also shown.

The binding affinity given as atomic contact energy (ACE) value for 3',4'-dihydroxyflavone and the standard ligand GABA at GABA_A_ (α_2_ subunit containing) receptors are shown in Table 2. In silico studies revealed a good binding affinity for 3',4'-dihydroxyflavone (-197.13 Kcal/mol) at GABA_A_ (α_2_ subunit containing) receptor when compared to standard ligand GABA (-93.36 Kcal/mol). A different binding pose predicted for 3',4'-dihydroxyflavone through the following amino acid residues (Phe 127, Phe 128, Gly 131, Lys 132, Lys 133, Ser 134, Gly 185)when compared to the standard ligand GABA (Asp 204, Ser 205, Val 206, Leu 220, Leu 221, Gly 222, Glu 223, Ser 224, His243, His245) at GABA_A_ (α_2_ subunit containing) receptors (Figure 8A and 8B).The standard ligand GABA forms two H-bond interactions at the following amino acid residues Leu220 and Gly222.In case of 3',4'-dihydroxyflavone, the amino group of the residues Lys132, Lys133 forms H-bond interaction at 3'-position of the side chain. Another H-bond interaction is formed between the keto groups at the4th position of flavone nucleus with the Ser 134 residue. The ACE value of ligands such as pinacidil and 3',4'-dihydroxyflavone at K_ATP_ channel are shown in Table 2. In silico studies showed a good binding affinity for 3',4'-dihydroxyflavone with an ACE value of -246.94 Kcal/mol at K_ATP_ channel comparable to the standard ligand pinacidil (-273.08 Kcal/mol). The following amino acid residuesLys48, Lys49,Asn50, Gly51, Lys189, Arg190, Gln192, THR193, Ser197, Arg214, Arg313, Ser314 and Tyr316 may be predicted to be important in the binding interactions of the standard ligand pinacidil at K_ATP_ channels (Figure 8C and 8D).Docking study revealed similar binding pose for 3',4'-dihydroxyflavone that forms key binding interactions at K_ATP_ channels. The standard ligand pinacidil does not form H-bond interactions with the amino acid residues mentioned. In case of 3',4'-dihydroxyflavone, H-bond interaction is formed between the 3'-position of the side chain of flavone nucleus and the Asn50 residue. The ACE value of ligands such as adenosine and 3',4'-dihydroxyflavone at adenosine (A_3_) receptors are shown in Table 2. Docking studies showed a good binding affinity for 3',4'-dihydroxyflavone (-250.42 Kcal/mol) at adenosine (A_3_) receptors comparable to the standard ligand adenosine (-283.97 Kcal/mol). The standard ligand adenosine forms important binding interactions with the following residues Ala 69, Val 65, Met 66, Val 71, Leu 68, Val 72, Ser 73, Ile70, Tyr 265, Thr87, Leu84 and Met86. Docking of 3',4'-dihydroxyflavone predicted a binding pose similar to the standard ligand adenosine at adenosine (A_3_) receptors (Figure 8E-8F). Adenosine forms two H-bond interactions at the following residues Tyr265 and Met86. In case of 3',4'-dihydroxyflavone, H-bond interactions occur with the following amino acid residues Ser73 and Tyr265.

## Discussion:

CIPN is the most prevalent neurological complication of commonly used first line cancer chemotherapeutic drugs. It manifests as severe pain involving sensory deterioration with long-term functional impairment affecting the quality of life of patients. Though several compounds including anticonvulsants, antidepressants, opioids and other topical agents have been investigated for their efficacy in CIPN, satisfactory resolution of the menacing symptoms is still elusive. Earlier studies have reported the efficacy of gabapentin in the prevention of peripheral neuropathy in cancer patients treated with paclitaxel [21]. However, the occurrence of severe adverse effects like somnolence, ataxia and convulsions restrict the therapeutic efficacy of gabapentin [10]. Hence, there is an imminent need to identify a new compound to treat CIPN effectively. Many novel compounds including flavone derivatives are being explored for their beneficial effect in CIPN. In an earlier study, 3',4'-dihydroxyflavone demonstrated significant attenuation of acetic acid induced abdominal constrictions (visceral pain) in mice [12]. This report prompted an investigation on the prospective anti-neuropathic effect of 3',4'-dihydroxyflavone in mice model of paclitaxel induced neuropathy.

The behavioural parameters such as tactile allodynia, cold allodynia and thermal hyperalgesia were clearly evident on the next day after paclitaxel (10 mg/kg, i.p.) administration in mice[11, 16].The findings of the present study revealed significant amelioration of paclitaxel-induced neuropathic symptoms in all the behavioural parameters tested after treatment with different doses of3',4'-dihydroxyflavone. The standard drug gabapentin significantly attenuated the neuropathic symptoms induced by paclitaxel in mice. In Von Frey's test, animals treated with 3',4'-dihydroxyflavonedemonstrated significant reduction in the paw withdrawal response score in mechanical allodynia compared to paclitaxel treated animals. In acetone test, the paw withdrawal response score was significantly decreased with 3',4'-dihydroxyflavone treatment compared to the paclitaxel treated mice revealing its ability to attenuate cold allodynia. In hot water tail immersion test, significant increase in the reaction time to flick the tail was recorded in a dose-dependent manner indicating the anti-hyperalgesic effect of 3',4'-dihydroxyflavone.Thus, the present findings explicitly reveal the anti-neuropathic effect of 3',4'-dihydroxyflavone in paclitaxel model of peripheral neuropathy in mice. This is in agreement with previous reports on the anti-neuropathic effect of various flavone derivatives in paclitaxel model of peripheral neuropathy [11, 22, 23, and 24].

The pathophysiological mechanisms such as mitochondrial dysfunction, oxidative stress and microtubule damage have been implicated in paclitaxel induced nerve injury which is followed by inflammation and alteration in the ion channel activity leading to peripheral neuropathy [1, 3, 25 and 26]. Several flavone compounds targeting the aforementioned pathogenesis have been investigated for their efficacy in preventing CIPN. However, studies on flavone derivatives targeting the neurotransmitter function and the ion channels especially the K_ATP_ channels in the prevention of CIPN are sparse. Hence, investigations on the involvement of these mechanisms in the action of 3',4'-dihydroxyflavone was considered by employing suitable interacting chemicals.

Modulation of subtype selective GABAergic system is considered to be effective in the prevention of CIPN. Moreover drugs that enhance GABAergic neurotransmission such as pregabalin and gabapentin are commonly used in the treatment of different categories of neuropathy [27].The findings of the current study revealed a complete reversal of the protective effect of 3',4'-dihydroxyflavonewith bicuculline pre-treatment against paclitaxel induced neuropathic manifestations. Earlier studies have reported the anti-neuropathic effect of synthetic flavone compounds involving the a2 subunit containingGABA_A_ receptors [11, 13]. Thus, the present observation clearly demonstrates that the modulation of GABA_A_ receptors plays an important role in the anti-neuropathic effect of 3',4'-dihydroxyflavone. The limitations of the present study was that it did not employ flumazenil (GABA_A_ antagonist) to investigate the involvement of benzodiazepine site of GABA_A_ receptors in the anti-allodynic effect of 3',4'-dihydroxyflavone. Studies have reported that paclitaxel administration increased the incidence of neuronal excitability by causing alteration in expression of K+ channels in cortical and dorsal root ganglion (DRG) which results in the development of CIPN [28, 29]. Hence, activation of this channel with K_ATP_ agonists can be used to prevent peripheral neuropathy. Various compounds have been shown to involve K_ATP_ channels in the anti-neuropathic effect against paclitaxel induced peripheral neuropathy [14]. Hence, it was considered interesting to evaluate the involvement of K_ATP_ channels in the anti-neuropathic effect of 3',4'-dihydroxyflavone in paclitaxel model of neuropathy. The results of the current study revealed significant reversal of the neuroprotective effect of 3',4'-dihydroxyflavone with glibenclamide pre-treatment in paclitaxel induced neuropathic behavioural parameters. Thus, the present observation explicitly indicate that, activation of K_ATP_ channels plays an important role in the neuroprotective effect of 3',4'-dihydroxyflavonein the mouse model of paclitaxel induced neuropathy. The present observations are in accordance with the earlier reports on the neuroprotective effect of flavone derivatives mediated by activation of the K_ATP_ channels [11]. However, the effect of 3',4'-dihydroxyflavone on K_ATP_ channels may not be exclusively responsible for its protective effect in neuropathy since activation of other types of K+ channels have also been implicated in obtunding neuropathic pain [30]. Future studies may throw more light on this aspect and the complete effect of 3',4'-dihydroxyflavone on different types of this important neuronal inhibitory ion channel may be understood in its full perspective. Recent evidences suggest that, purinergic pathway including adenosine and their receptors play an important role in the prevention of neuropathic pain in preclinical models. It has become obvious that the adenosine receptor subtypes (A_1_, A_2A_, A_2B_ and A_3_) play an active part in the anti-nociceptive effect of different compounds in various pain models especially neuropathic pain [31]. Despite a potent anti-nociceptive effect reported with adenosine (A_1_ and A_2_) agonists in different pre-clinical models of neuropathic pain, the appearance of cardiovascular side effect has limited their clinical use. Recent studies have demonstrated the expression of adenosine A_3_ receptors in microglia, astrocytes and oligodendrocytes that are involved in the development of tactile allodynia [32]. Hence, selective stimulation of adenosine A_3_ receptors has shown prolonged anti-allodynic effect in chemotherapy induced and other models of neuropathic pain [33]. The findings of the current study revealed significant reversal of the neuroprotective effect of 3',4'-dihydroxyflavonewith caffeine pre-treatment in mice model of paclitaxel induced neuropathic manifestations and thus substantiate the participation of adenosine receptors in this neuroprotective effect. The present observation is in accordance with previous reports involving adenosine receptors in different neuropathic pain models .Paclitaxel treated animals exhibit an increase in the pro-inflammatory cytokines such as TNF- α and IL-1βin the DRG neurons, astrocytes and microglia leading to severe neuropathic manifestations. Recent studies have reported a decrease in the release of pro-inflammatory cytokines mediated by stimulation of A3receptors.Pre-clinical studies on flavonol and its dimethoxy derivatives demonstrated an inhibitory effect in the release of pro-inflammatory cytokines in paclitaxel induced peripheral neuropathy. It may be suggested that, the neuroprotective effect of 3',4'-dihydroxyflavone in paclitaxel treated mice may also be due to a decrease in the pro-inflammatory cytokines mediated through adenosine receptors. However, this has to be substantiated by future studies.

## In silico studies:

Molecular docking studies were carried out to identify and predict the binding sites of 3',4'-dihydroxyflavone at the investigated targets for mechanism of action. The interaction of 3',4'-dihydroxyflavone with the binding sites on human GABA_A_ (α_2_ subunit containing) receptors, K_ATP_ channels and adenosine A_3_ receptors were analysed mechanistically. In silico studies predicted a good binding affinity for 3',4'-dihydroxyflavone at GABA_A_ (α_2_ subunit containing) receptors based on interaction energy value compared to the standard ligand GABA (Table 2). The predicted binding sites of 3',4'-dihydroxyflavone through H-bond interactions at GABA_A_ (α_2_ subunit containing) receptors were different from those of endogenous ligand GABA (Figure 8). Hence, it may be predicted that 3',4'-dihydroxyflavone exerts anti-neuropathic effect by binding to an allosteric site on GABA_A_ (α_2_ subunit containing) receptors. The significant interaction energy noted for 3',4'-dihydroxyflavone at K_ATP_ channels indicates a good binding affinity for this flavone derivative compared to the standard ligand pinacidil (Table 2). The binding pose predicted for 3',4'-dihydroxyflavone at K_ATP_ channels through H-bond interactions was almost similar to the reference drug pinacidil (Figure 8). Molecular docking studies also revealed a good binding affinity for both endogenous ligand adenosine and 3',4'-dihydroxyflavone with similar ACE values at adenosine A_3_ receptors (Table 2). Docking of 3',4'-dihydroxyflavone at adenosine A_3_ receptors predicted a similar binding pose like the endogenous ligand adenosine through H-bond interactions (Figure 8). The results of the present study on 3',4'-dihydroxyflavone revealed good binding affinity with excellent binding pose at the selected receptor targets. The observations of the molecular docking study corroborate the results observed in in vivo experiments in the mouse model of CIPN. The involvement of GABA_A_ (α_2_ subunit containing) receptors, K_ATP_ channels and adenosine A_3_ receptors in the action of 3',4'-dihydroxyflavone has been unambiguously substantiated by molecular docking studies.

## Conclusion:

 Data shows the anti-neuropathic effect of 3',4'-dihydroxyflavone against paclitaxel induced peripheral neuropathy in mice by effectively reducing different neuropathy behaviours. Moreover, the results indicate the involvement of GABA_A_, K_ATP_ and adenosine receptors in the ameliorative effect of 3',4'-dihydroxyflavone in CIPN. Potent anti-neuropathic compounds with minimum adverse effects are needed to alleviate the clinical manifestations of CIPN which is highly refractory to current treatment regimen. Further investigations on the effect of 3',4'-dihydroxyflavone in CIPN due to other chemotherapeutic drugs shall further strengthen the potential benefits of this flavone.

## Figures and Tables

**Table 1:** Study design for investigation of mechanism of action of 3', 4'-dihydroxyflavone

**Day 1**	**Day 2**
0 min	15 min	45 min
Paclitaxel 10 mg/kg; i.p	Behavioural assessment Vehicle/ Interacting drug	Vehicle / 3',4'-dihydroxyflavone	Behavioural assessment

**Table 2:** Molecular docking: Binding affinity (Atomic contact energy, ACE) score of 3', 4'-DHF and standard ligands at GABA_A_ (α_2_ subunit), K_ATP_ and adenosine (A_3_) receptor

**Compound**	**ACE value at GABA_A_ (α_2_ subunit) Kcal/Mol**	**ACE value at K_ATP_ Kcal/Mol**	**ACE value at Adenosine (A_3_) Kcal/Mol**
3',4'-dihydroxyflavone	-197.13	-246.94	-250.42
GABA	-93.36	-	-
Pinacidil	-	-273.08	-
Adenosine	-	-	-283.97
